# Factors Influencing Retirement Decisions among Blue-Collar Workers in a Global Manufacturing Company—Implications for Age Management from A System Perspective

**DOI:** 10.3390/ijerph182010945

**Published:** 2021-10-18

**Authors:** Ellen Jaldestad, Andrea Eriksson, Philip Blom, Britt Östlund

**Affiliations:** 1 Department of Biomedical Engineering and Health Systems, KTH Royal Institute of Technology, SE-141 57 Huddinge, Sweden; andrea4@kth.se (A.E.); brittost@kth.se (B.Ö.); 2 Scania CV AB, Health & Work Environment, 8000 AP Zwolle, The Netherlands; philip.blom@scania.com

**Keywords:** international study, job crafting, older workers, prolonged work life, sustainable work

## Abstract

The maintenance of older workers and determining the appropriate age for retirement are growing issues related to the fact that fewer people, still active in working life, have to provide for more non-working people due to increased life expectancy. As a result, retirement age has started to rise in many countries, and employers need to find ways to maintain an older and healthy work force, not least to avoid the loss of important experience. The aim of the current study was to increase the knowledge of factors influencing the retirement decisions among blue-collar workers in different national settings. A survey and semi-structured interviews were conducted with a sample of 100 blue-collar workers in Sweden, the Netherlands, and France, aged 55 years and older, within a global manufacturing company. Based on the results, implications for companies’ age management strategies were discussed from a system perspective. Factors contributing to both retirement and to a prolonged work life were found on individual, organisational, and societal levels. This indicates the importance of a system perspective when planning for age management interventions.

## 1. Introduction

As the average life expectancy in the western world continues to increase, the possibility of continuing to work also increases for those who have health and motivation. Socioeconomic factors and work conditions such as physical and psychological strain impact aging employees’ possibilities and/or individual motivation to retain more years at work. Today, we know quite well what is influencing retirement [1], but the problem is the lack of knowledge and age management strategies on how to influence decisions to postpone retirement. Age management can be defined as taking the employee’s age and age-related factors into account in daily work management, work planning, and work organisation, thus enabling everyone, regardless of age, to achieve personal and organisational targets healthily and safely [2,3].

The decision to leave the labour force is a complex one including a range of factors that need to be considered in a company’s age management. A poor physical work environment tends to influence employees’ motivation to retire early. So do psychosocial factors, the organisation of work, and the content of job tasks, especially the degree of influence at work and the opportunity to pass experiences on to younger generations [1,4,5,6,7,8,9,10,11], as well as ill health and disabilities due to musculoskeletal disorders [12,13,14]. Other considerations that affect retirement decisions concern financial incentives for retirement, e.g., uncertainty regarding being able to provide for the household without a full income [14,15,16,17], as well as family and social life outside work. Family members’ retirement also increases the risk of early retirement [6,14,18]. Opportunities for development and influence at work, responsibility for others, and satisfaction with working hours and meaningful tasks reduce that risk [6,8,14,17,19].

There are indications that continuing working has a positive relation to health [20] and early retirement has negative health effects [21]. Good health has also been shown to be an important factor for positive attitudes towards job retainment [8]. The duration of working life varies between company branches and between men and women. Research points out that some groups are overrepresented in terms of working longer in old age, especially farmers, while knowledge about retirement age for other businesses and for employees in technology companies is lacking [2]. Manufacturing is one of the strongly overrepresented industries in the labour market in terms of reported occupational accidents and diseases, physical disorders, and fatigue [22,23].

There is also limited research on how organisations can support and motivate older employees to continue to work beyond retirement age, but existing results point to the fact that employability and productivity can be maintained much further than we would normally expect [5,24]. To implement, and focus, age management is beneficial, regardless of sector and size of the organisation [25]. Bottom-up employee-driven job crafting interventions for older employees have in this context been advocated, because they have been shown to increase motivation for prolonged working life [20] as well as increase production and decrease absenteeism [26]. The concept of job crafting can encourage employees to reshape and improve the fit between the characteristics of the work and their own needs, abilities, and preferences [27,28]. Three types of crafting related to age management were published in Pit, Shrestha, Schofield, and Passey [13]; new skills to prevent jobs becoming monotonous, interpersonal relationships, and employees’ cognitive stance toward their work by positively reframing the manner in which they think about aspects of the job. Other studies confirm that job crafting interventions in general working populations may increase individuals’ well-being and health [29,30] with an even bigger effect on older workers [31].

However, there is also a need for organisational structures and management support for implementation of age management interventions. To educate managers to be ’age aware’ and to establish an environment that enhance the workability and effort of older employees, e.g., developing strategies for mentoring and age management education for managers, can in this context be seen as important [32]. An environment supporting and encouraging older employees to keep on developing and to actively counteract age discrimination was shown to be especially important for work engagement and affective commitment to work and the employer [33].

**Figure 1:**
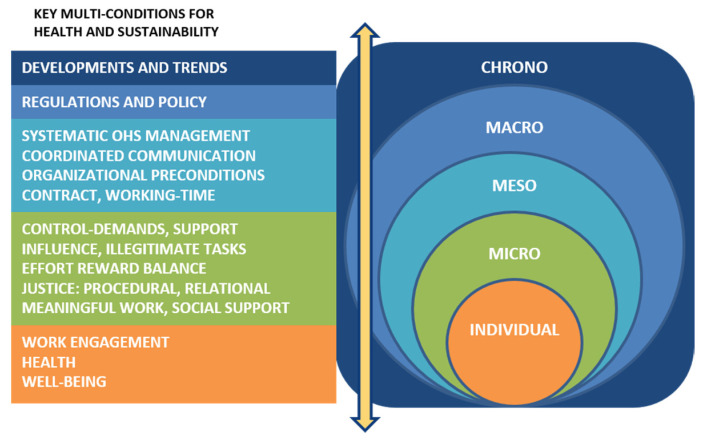
Examples of aspects on the different system levels included in the study. The arrow indicates the interplay between the levels (modified from [35]).

Overall, research reviews have shown that age management interventions yielded better results if they included more than one organisational dimension than if they focused on one aspect [34]. It is therefore suggested that combined measurements on different organisational levels can support consistent results rather than interventions merely directed towards individuals [24,35]. System perspectives have in decades been advocated for workplace health interventions [36] and have recently also been suggested for considering the inter-relationships between different factors impacting a prolonged working life [1]. However, this kind of system approach has rarely been adopted in age management interventions. Nilsson [24] presents the swAge-model, which argues the importance of an age awareness in working life. The model holds a system perspective on sustainable age management in which different forms of ageing (i.e., biological, chronological, social, and cognitive ageing) are related to areas important to older employees’ ability and willingness to continue working. Strategies to enable individuals to retain in work are, according to the model, formed by structures in both the organisational (meso) level and in the societal (macro) level. A system approach like the swAge-model allows for a holistic approach to different factors in interlinked subsystems, affecting the health and motivation of older workers to continue working. System models commonly contain structures on different levels, and the interaction between these subsystems are highlighted [35,37,38]. This study was based on a system model including a time perspective (chrono), a societal level (macro), two different organisational levels (meso and micro), and one individual level [37], as presented below and in Figure 1. According to the swAge-model [24] employers need to take into account that their older employees age differently. They are, for example, differently affected by biological changes. Thus biological ageing interacts with aspects on the individual level; one employee might need more variation in work than another to be able to stay healthy at work. On the meso-level chronological ageing is, following the swAge-model [24], connected to personal economics through, for example, statutory retirement age; employees in one country may have to work longer than their colleagues in other countries. On the macro- and micro-levels, organisational and manager attitudes towards older workers are related to social ageing in the swAge-model [24], and can influence willingness to stay in work. Examples of aspects that, according to the swAge-model, are connected to a person’s cognitive ageing include opportunities to participate in professional development and work satisfaction [24]. These aspects are found on the micro-level and on the individual level, respectively. Thus, many aspects interrelate on different levels, as well as with the individual’s ageing process. This indicates the need for adjusting work to the individual worker, from their current state of health.

The chrono-system level is a time factor and is here described as, for example, trends in society that may have importance for workers’ health and wellbeing, such as political decisions and changing pension regulations [35]. A rising life expectancy is also included in the chrono-level, as well as the different forms of ageing presented above [24]. The macro-system level includes policies and legislation and can in this context be the national retirement legislation that companies need to consider and follow. The meso-system level is manifested by organisational structures and organisational culture and includes health and safety management systems, company policies related to age management, and organisational preconditions for age management. The micro-system level is directly experienced by the employees and is where interactions between colleagues, and between managers and subordinates, occur. Work demands, resources, control, and social support are manifested at the micro-system level. The individual level includes individuals’ handling strategies related to work and identity, motivation, health, and wellbeing. The individual level also holds a person’s knowledge, capability, attitudes, values, lifestyle, and behaviour [35].

In conclusion, an integrated system model for how employee wellbeing could be crafted through managerial work and managerial strategies can be applied to analyse preconditions for more holistic age management interventions and implementing those [24,35]. The application of this kind of system approach may facilitate a work organisation’s understanding of the complex and interrelated factors influencing a worker’s retirement decision [1]. To our knowledge, there is no previous study investigating factors contributing to blue-collar workers’ retirement decisions from a system perspective, conducted in a global manufacturing organisation. The aim of the study was, thus, to increase the knowledge of factors influencing the retirement decisions among blue-collar workers in different national settings. This was done from a system perspective, and implications for companies’ age management strategies was discussed accordingly.

## 2. Method

### 2.1. Company Presentation and Pension Systems

Scania CV AB, with its head office in Sweden, was founded in 1891 and is now a worldwide provider of transport solutions, including trucks and buses for heavy transport applications. In 2017, the company had around 49,300 employees in about 100 countries. Production takes place in Europe, Latin America, and Asia.

The company is working actively with age management with the aim to enable blue-collar workers to continue working longer, with retained health and work capacity. The company has an internal occupational health service, which aims to work proactively; developing a safe, healthy, inspiring and productive workplace that rewards good performance. Representatives of occupational health service connect the work with age management to one of the company’s core values ‘respect for the individual’; meaning the individual stands at the centre in all aspects.

The blue-collar workers in this study worked on different kinds of workshop tasks. Most of them worked on the assembly line, putting different parts of a truck in place on a fixed time cycle. They worked in teams and rotated regularly between different workstations during the day. Some respondents worked in stations outside of the assembly line, for example in painting and in engine testing, or assembled the final parts of the vehicle. Those stations were not on a fixed time cycle. Some respondents worked in logistics within the production plants. They were able to plan their day largely than the other groups of respondents. Among the tasks was also quality control, which was considered as a ‘calmer’ work task, where, for example, assembled parts on a vehicle were controlled. Other ‘calmer’ tasks, such as cleaning, had earlier been available for older workers or people on rehabilitation. However, those tasks were now outsourced or included in other work tasks, reducing the opportunities for older employees to work at a slower pace and with a less heavy physical workload and more autonomy.

Acting on a global arena, the company is affected by national and local pension schemes in the different countries (see Table 1).

### 2.2. Data Collection

Data was collected during 2016 and 2017 and included a sample of 100 blue-collar workers aged 55 and older in Sweden (*n* = 29), the Netherlands (*n* = 37), and France (*n* = 34). Respondents were selected within five production plants; three in Sweden, one in the Netherlands, and one in France. The data collection was mainly conducted using a survey, including factors previously shown as contributing to retirement, distributed to all blue-collar workers in the sample, and semi-structured interviews with all blue-collar workers in the sample with the aim to provide a deeper understanding of the survey data.

The respondents included full-time workers, part-time workers, and full-time retired workers. All respondents had been employed as blue-collar workers at the company for at least the latest four years (currently employed or before retirement). The retired respondents had all retired within the last 12 months. Respondents were asked to participate voluntarily by selection from the oldest person available and downward in age (see Table 2).

A computerized survey, based on previous research in the area (see Table 3), was conducted individually guided by either an internal or an external interviewer. The internal interviewers worked either in HR or in the occupational health and safety department. The external interviewers were the first author of this article, one HR-student, and one HR-trainee. In Sweden and in the Netherlands there were both internal and external interviewers. In France, one external interviewer conducted the interviews. The first author of this article trained all interviewers. The survey was open for comments and followed by the semi-structured interview with open-ended questions. All interviews were held in the first language of each country (Swedish, Dutch, and French) by a native-born interviewer.

A pilot study was initially conducted with respondents in Sweden and in the Netherlands, to test and adapt the survey questions and the interview guide to the study settings. During this phase, interviews were also held with trade union and workers council representatives, as well as HR partners in all countries, which resulted in some adding of interview questions. All questions were professionally translated from Swedish into Dutch and French and thereafter discussed within the project group of each country to ensure that the questions were perceived in the same manner in the different cultural contexts. This was a process that included many steps back and forth between the researcher team and project teams, to ensure a proper translation and mutual understanding of the questions included in the survey and in the interviews with the blue-collar workers. In this process, some aspects were removed from the French version of the survey, e.g., “sense of coherence”, and “experience of ageism”. The reason for this was that the French project group was not able to find a suitable formulation of the aspect, or considered the aspect too personal to ask in a work setting.

During the study, the first author of the article, to gain an understanding of the work environment and work settings for the older workers, visited all five production plants. Notes were taken during an integrative walk through with company representatives. To complete the data collection the first author of the article was provided with some internal personnel information, such as age and gender distribution in the different production plants and countries. This data, and the observations of the plants, were not analysed.

The Ethical Review Board in Stockholm approved the study and informed consent was applied; all respondents were informed verbally and in written text about the study before accepting participation. The respondents were also informed that the interview could be stopped at any time, with no further questions asked and that no data would be kept. All material was handled confidentially within the company and coded when reported back to the first author of the article, to ensure anonymity.

In the survey, the respondents were asked to take a stand on different aspects of given factors shown by previous research to influence retirement decisions (see Table 3 for item examples). The respondents were asked to state whether the aspects contributed to retirement or motivated them to prolong work life, with the stem questions “Which of the following aspects are crucial when you consider retirement?”, and “In what way are the chosen aspect(s) crucial—Do they contribute to retirement or to a prolonged work life?” The term “retirement” included both “early retirement” (retirement before statutory retirement age), and “retirement on statutory retirement age’. All questions were open for comments. In the semi-structured interview, the respondents were, for example, asked about how to improve the work environment for older employees, and if they considered other aspects than the ones in the survey to be crucial for their decision about retirement; e.g., “What changes would you like to see at your job that can contribute to a better work environment for older workers?” and “Beyond the aspects you previously took a stand on, are/were there any other aspects important to your decision regarding retirement?”.

### 2.3. Analysis

The results from the survey were first descriptively organized into percentages of respondents in the different countries who stated the aspects of the different factors either as contributing to retirement or to a prolonged work life, or if the aspect had no impact on the decision. If the respondent had stated that the aspect was important, but not commented in what direction, the interview answers were used to understand in what way the aspect was considered as important. Secondly, the semi-structured interviews, as well as the comments in the survey, were used in order to gain understanding and give substance to the individual answers from the survey. In all, the analysis of the empirical data was conducted in three steps;

1. The survey data indicated if the aspect was crucial or not to the decision about retirement.
2. The comments in the survey were used to code in what direction the aspect was considered as crucial; to retirement or to a prolonged work life.
3. The individual answers from the interviews were used to understand in what way the aspect was considered as crucial in the present context, e.g., “shift work is hard, especially night shifts, and has been so for at least ten years” (blue-collar worker in Sweden), and “the opportunity to work daytime is the main reason for me to be able to continue working” (blue-collar worker in the Netherlands).

The qualitative data were further analysed from the themes in the survey. This meant that how similarities and differences in between different individuals (e.g., the individuals’ current health status), different workplace contexts (e.g., support from colleagues and superiors), and different national contexts (e.g., local pension systems) influenced the perceived importance of an aspect was analysed. The qualitative data thus deepened the understanding of why and in what ways a specific aspect was important to the respondents. It also showed how aspects on different system levels interacted in the respondents’ decision about retirement. The qualitative data were therefor used in the development of the proposed system model and intervention activities discussed below. To summarize and visualize the results, we used the system model presented in the introduction. There were aspects considered crucial to the blue-collar workers’ decision about retirement in all system levels except the chrono-level. The chrono-level was not used in the survey, nor in the analysis. The chrono-level was, however, used when discussing the results.

## 3. Results

In this section, results that represent differences between the countries, as well as the interplay between different system levels, are presented. This is done in order to lay the foundation for the intervention actions discussed below. In Table 4, we summarize what we consider the most important findings on the different system levels, as well as the main differences between the countries. All of the descriptive results from the survey are presented in Table 5. The table holds the percentages of aspects considered as crucial to either retirement or to a prolonged work life, or as having no effect on the decision. This is presented for each country and in total. 

### 3.1. Macro Level

(Laws and regulations; e.g., national retirement legislation, culture and values, and attitude towards an older workforce)

In both Sweden and the Netherlands, the national pension system was considered tough. According to the comments, the respondents tried to ‘adapt the size of the wallet’ and use personal savings to handle the upcoming changes in their personal economy. The respondents also talked about a general view that older people should ‘leave room for the younger ones’, indicating a negative perception of the attitude towards an older work force in the society.

### 3.2. Meso Level

(Policies and company culture, attitude towards an older work force, local pension agreements, strategic management and communication, and working hours)

On the meso-level, within the factor work organisation, the setup of working shifts (28%) was the most crucial aspect to retirement, whilst work distribution (21%) and setup of job rotation (13%) were important to prolonging work life.

The aspect setup of working shifts as contributing to retirement was most prominent in the Netherlands (48.6%) compared to Sweden (20.7%) and France (11.8%). Shift work in general, and night shift in particular, were considered tough and were said to highly influence the decision regarding retirement. Working extra hours (e.g., ‘flex-time’) and on weekends had a negative impact, and some respondents requested to work daytime only. However, the work distribution and setup of the working hours and working shifts were also important aspects to consider for a prolonged work life, since shift work was mentioned as a positive aspect due to enhanced variation. Setup of work and breaks, and job rotation were also considered positive factors.

Regarding the local pension agreement, 41.4% of the Swedish respondents said they had, or would have, enough pension to provide an income without working past retirement age. The local pension agreement in Sweden was mentioned positively among several of them.

### 3.3. Micro Level

(Psychosocial work environment, responsibility and influence, management approach, attitude towards an older work force, and a physical work environment)

Within the factor of physical work environment, physical workload (30%), repetitive work (20%), and (lack of) variation and recovery (16%) were the most prominent aspects considered crucial to decisions about retirement. Heavy workload and repetitive tasks, as well as not being able to sit down during work, were said to be tiring and hard for the older workers. Some respondents said they had had injuries due to the physical work and some said that noise, worse lighting, vibrations, and working in uncomfortable positions could, or had, affected their decision about retirement. Variation and recovery (20%) was considered as an important aspect to prolong work life, especially in the Netherlands where 32.4% percent of the respondents had this opinion, as well as to have influence and responsibility for others (15.2%; France excluded), and to be appreciated by others (20%),

In total, 13% of the respondents considered the availability of ergonomic tools and safety equipment as important to prolonging work life. In Sweden the corresponding number was 3.4%, and comments were that ergonomic tools were not available. Workers on the assembly line said they were very tired after work and needed to rest to recover during evenings and weekends. They did not want their reduced energy levels to affect work, but it affected their spare time and their social life outside work more than ever. A gradual transition to retirement was therefore requested to reduce the physical workload.

Within the factor of psychosocial work environment, work-related stress (34%) and demands and control (22%) were the most prominent aspects crucial to retirement. These were followed by the atmosphere and comfort at work (17%) and support from closest superior (15%). Some respondents expressed that the stress had increased; ‘…more is to be produced with the same staff’, and the older workers were not able to keep up with the pace on the assembly line. In France, the atmosphere (38.2%) and support from the closest superior (26.5%) were mentioned as factors contributing to retirement to a greater extent than to a prolonged work life (14.7% and 8.8%, respectively), with respondents commenting that they lacked support, recognition, and respect from their closest superior, and that the pressure had increased. Comments were that the closest superiors were ‘too busy fulfilling their personal goals’ instead of managing their group. Superiors were also said to have a lack of understanding of the challenges the older workers faces, as well as of how to make use of older workers’ competence.

The atmosphere and comfort at work (35%) and support from closest superior (23%), along with social support from colleagues (28%), were also the most important aspects for a prolonged work life within the same factor. Social activities outside work, to talk to and get to know one’s colleagues, as well as the possibility to plan their own work day, were said to have a positive effect on the amount of work-related stress and on the balance between work demands and control. Among the respondents who considered these aspects important to prolonging work life were those who said they actively tried to improve relations with both colleagues and supervisors, for example by socialising with colleagues during the day and developing a trusting relationship with their managers. The respondents in the Netherlands considered most of the aspects of the psychosocial work environment as important to prolong work life (e.g., social support from colleagues and superiors, cooperation, and the atmosphere at work) whereas the respondents in France mostly considered the same aspects as contributing to retirement.

There were different experiences of the attitude towards an older work force within the company among the respondents; some said they had not noticed anything regarding the attitude, whereas some had experienced only, or almost only, positive attitudes among colleagues and supervisors. Some said negative attitudes and comments at work due to age occurred. Even though most respondents thought it was positive to work in mixed groups with different ages, genders and cultures, some felt excluded by the working group due to their age.

### 3.4. Individual Level

(Economic situation, health and meaningfulness, competence and experience, engagement and belonging, and personal effort)

To experience work as interesting and meaningful (21%) and to have suitable competence to conduct work (12%) were important aspects for a prolonged work life within the factor ‘job task characteristics’. However, in the Netherlands neither of these aspects were considered important in any direction by more than 11% of the respondents, and in France the same amount of respondents (23.5% respectively) considered interesting and meaningful job assignments as important to retirement as to a prolonged work life.

The opinions in all countries also differed between those who said their work was interesting and meaningful and those who said they wished they had had more opportunity to develop within their work. Among the respondents who considered the job task characteristics meaningful and interesting, and also important to prolonging work life, there were those who said they had the opportunity to change and develop work to better suit their personal goals and needs, and to adjust work in discussions with colleagues and supervisors, for example in weekly quality meetings. Contrasting to this, those who considered the aspects contributing to retirement said work was too strict and standardised, leaving no room for individual changes. According to the comments, more development opportunities would have had contributed to a prolonged work life. In France some did not consider work as adding meaning to life; “work is work and not what gives life meaning”.

In terms of personal effort, 14.7% of the French respondents said that being content with one’s own effort influenced their decision about retirement. The same amount of respondents also said that being able to use one’s competence was crucial, indicating in their comments that they used to be more content with work some years ago but that different aspects, such as more pressure and a different atmosphere, had changed that view. In total, to be content with one’s own effort (23%) and to find the job meaningful (19%) were the most important aspects to a prolonged work life within the factor of “job task characteristics”. Many respondents were proud of their jobs, and of the company. The personal mind-set and motivation were said to be important for being content with one’s own work.

Many of the respondents said they had to keep working to retirement age or beyond in order to provide for basic needs (43%) and/or to maintain their current lifestyle (15%). This was largely seen in the Netherlands and in France, compared to Sweden. As mentioned above, personal savings were said to be a way to enable retirement, as well as to ‘adapt to the size of the wallet’ and to ‘plan one’s spending’ more carefully. Even though the economic situation affected many respondents in their retirement decisions, some said ‘there are more important factors,’ such as health and spending time with family and friends.

Current health (45%) as well as future expected health (39%) were among the most crucial reasons contributing to retirement in the entire survey. Among the comments were: ‘It is important to have a healthy life when retired’, and ‘I want to retire with my health left intact’. There were, however, also respondents who said they already had diseases and injuries to cope with, but that they would rather go to work than stay at home; ’It is better to work than to sit at home’.

Respondents who had experienced a life crisis or sickness themselves, or among family and friends, said this affected their decision to retire as early as possible. Having good health—current and expected—also influenced their decision to prolong work life; to work helped the respondents to feel well.

Among personal factors, to spend more time with family, friends, and on leisure activities was the most prominent aspect towards retirement (41%). There were also those who said they wanted to retire because of this aspect, but that they were not financially able to. According to open answers and comments, partners’ retirement influenced the decision in different directions; there were those who said they wanted to keep working because their spouse was younger and those who wanted to stop working at the same time as their older spouse. However, there were also those who said they had to keep working because of their economic situation even though their partner was already retired. Overall, to enable a gradual transition to retirement was recurrently requested, in order to be more prepared for the upcoming changes in social life and in general life structure.

## 4. Discussion

**Figure 2:**
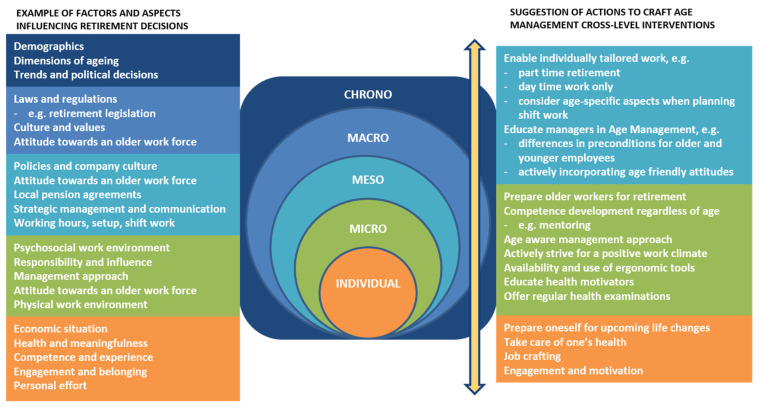
Examples of important aspects and age management actions suggestions based on the results. The arrow indicates the interplay between the levels (modified from [35]).

The aim of the study was to increase the knowledge of factors influencing the retirement decisions among blue-collar workers in different national settings. This was done within a global manufacturing company. The results of the study found factors influencing the retirement decision on different system levels. In this particular study we chose to look at the results from the following levels; chrono- (time factor), macro- (societal level), meso- (organisational level), micro- (work group/department level), and individual level (see Figure 1). In the following section, the results are compared and contrasted to previous research from those system levels. Based on the results, age management intervention actions are also discussed from a system perspective (see Figure 2).

On the macro-level (e.g., policies and legislations on the national level), aspects said to be important to the respondents’ decision about retirement included national retirement legislation. The statutory retirement age in the three countries in the study differed and global manufacturing companies thus need to consider that their blue-collar workers in some countries may have to work several years longer than in others. On the societal macro-level the culture and values in each country, as well as the attitude towards an older work force, might also differ. Among the respondents, there were those who said that the older workers should leave room for younger ones, even though most respondents considered the attitude towards older workers as positive. This aspect was not considered as influencing the decision about retirement largely; however, a global company should be aware of that when you are considered an older worker, a social aspect of ageing, might differ between countries [24].

An important aspect on the meso-level (i.e., organisational level) was access to the Swedish local retirement program. Several Swedish respondents in this study had access to the early retirement program, and most of them planned to take advantage of it, even though they said they felt healthy enough to continue working longer. Even though part-time retirement seem to have a positive outcome on keeping industrial employees in work beyond retirement age [40], agreements that enable people to stop working earlier may not encourage them to stay longer in the company [16,41]. However, such an agreement will likely be beneficial for the individuals’ health as pensioners. Age management interventions that include individually tailored work, such as part-time retirement, could be strategic to keep older workers and their competence within the company [24,40]. Among the respondents were also people with immigrant backgrounds who had not been living in Sweden long enough to be able to benefit from the local retirement program. Similar to the respondents in the other countries, where no such retirement program existed, they had to keep working longer. In France, comments regarding pension legislation included ‘I’ll stop when I´ve reached my quartiles’, indicating a possible social and cultural aspect of ageing, in not working longer than needed [24]. Age-specific aspects are also suggested to be considered in shift work planning [42], especially when non-senior older workers are involved. Due to, for example, multiple careers or emigration the older workers are not necessarily the most senior workers. Older shift workers who lack experience are probably the worst candidates working in non-traditional shift work [32].

The respondents had different ways to cope with work pressure and to gain and maintain energy, such as resting during evenings and weekends. They also talked about keeping healthy and having a positive attitude to work and life in general. Those are strategies on the individual level used by the blue-collar workers to handle challenges on other system levels. To reduce or eliminate the stressful work on the assembly line was suggested in all countries, as well as to work daytime only, to avoid flex work and over time, and to work shorter days. Even though shift work mostly was considered tiring, there were those who said shift work enabled variation, once again indicating the positive potential of more individualized work. In this study, the opinions of shift work differed the most between the Netherlands and France. In the Netherlands, where 48.6% of the respondents said shift work contributed to retirement, the average age of the respondents was 65 years. In France, the corresponding number was 11.8% and the average age of the respondents was 58 years. In France, the work shifts were also differently planned than in the Netherlands, and did not include overnight shifts. Both the age difference among the respondents and the fact that less respondents worked late shifts in France could explain why more respondents in the Netherlands than in France considered the aspect as contributing to retirement. To implement daytime work only may therefore not lead to the expected outcome in the different countries; the French workers may benefit less from such an implementation. These differences in the results also mirror the ambiguous findings of Blok and de Looze [42] who found differences between older and younger shift workers, but no evidence of more problems related to shift work among older workers. From the results of this study, a global company is also encouraged to strive for common values of older workers, and to consider how different aspects in the work environment affect those employees. This can be done by educating managers in age management and by developing strategies for internal communication to spread information within the organisation. Comments among the respondents regarding the attitude towards older workers in the company were overall positive, even though it was mentioned that the closest superior often lacked an understanding of the work situation for older workers. The attitudes towards older workers can differ on the different system levels, indicating the importance of having a common view on the meso-level to be spread through the organisation.

On the micro-level (i.e., workplace level) respondents in all countries asked for a gradual and smoother transition to retirement—to successively reduce the workload and to be more prepared for the upcoming changes in social life and in general life structure, which is also supported by the findings of, for example, Ilmarinen [5]. Mentoring younger and/or new colleagues can lead to learning and development as well as to more variation, skills transferring, and reduced workload for older workers, as well as to fully use the older workers’ competence. The respondents also indicated that their closest managers (e.g., team leaders) often were young and more focused on making a career of their own than to understand the challenges faced by older workers, e.g., they were ‘too busy fulfilling their own goals’. The understanding and interaction between managers and employees develop through time and attention, and are of importance in developing the company’s age management strategies [43]. To organise for mentoring and an age-aware environment on both the micro- and meso-system levels are in line with the suggestions of Nilsson [24], Popkin, Morrow, Di Domenico, and Howarth [32], van Dam, van Vuuren, and Kemps [33], Widell Blomé, et al. [44], and BestAgers [45], who all indicated the importance of including the older workers, as well as providing training regardless of age, and to educate managers in age management. By letting, and encouraging, older workers to participate in training and education, relevant tacit knowledge can also be made available to managers and younger colleagues [43]. To actively seek a positive working climate that takes ageing and cultural aspects of work into account is also indicated on the micro-level. Overall, the general development of a positive work climate can be seen as a facilitator of a prolonged working life [33]. The respondents were keen to keep a positive atmosphere at work and to enjoy social activities outside work. To work in mixed groups, with people of different ages, genders, and cultures were considered positive for the psychosocial work environment. Most of the respondents also said they were proud to work for the company; its brand had high value. Intrinsic job value is positively related to employability, work engagement, and affective commitment [33]. There was, however, a national difference in the survey, in which the respondents in France had a more negative view of both the atmosphere at work, and of the social support from colleagues and supervisors compared to the other countries. A cultural difference in how work was interpreted may be the reason for this. Comments from the French respondents were that the attitude within the company had changed over the years, as well as that work was not considered as adding meaning to life; ‘work is work, and not what gives life meaning’.

Availability of, and encouragement to use, ergonomic tools is an important aspect to reduce physical work load [24]. Some of the Swedish workers considered ergonomic tools and safety equipment to be an aspect contributing to retirement, whereas workers in the other countries considered this aspect as contributing to a prolonged work life. According to the Swedish respondents with this assumption, work was conducted with ‘less resources than before’ and featured ‘heavy load and lifting without ergonomic tools’. Since older workers benefit more from ergonomic tools [32], and since the production plants in the study were more or less identical, it is assumed that the respondents who considered ergonomic tools as contributing to early retirement experienced a lack of them. To enable alteration between physically and mentally demanding tasks it is thus suggested to increase the variation in work load [24,46]. Based on these findings, and in line with Lichtenthaler and Fischbach [20] and Loch, Sting, Bauer, and Mauermann [26], we propose a bottom-up management approach to involve the employees in the intervention process.

Age management interventions, regardless of which level they are planned on, aim to enable individual workers to stay healthy at work [3,47]. The individual worker is the key to realizing this. However, the organisation must in this context be responsible for developing preconditions for individuals to make healthy choices [24]. On the individual level the economic situation was the most important reason for the respondents to keep working; thus, the need to prepare oneself for the upcoming changes in life, especially the economic situation, is supported. The respondents talked about ‘adapting the size of the wallet’, and personal savings, but many of them said they had to keep working because they were not able to provide for their basic needs. Self-experienced health was one of the most important aspect in the survey to retire as early as possible, both current (45%) and expected (39%) health. According to Nilsson, Hydbom and Rylander [47] self-experienced health is a better predictor of retirement decision than diagnosed health. This indicates the importance of keeping the health factor in focus on all levels whilst planning a company’s age management strategies, as well as to encourage individual workers to take care of their own health e.g., by educating health motivators and providing regular health examinations with the occupational health care. The respondents also talked about being content with one’s own effort, and being able to use one’s competence and experiences in relevant ways. As mentioned previously, they wanted to be involved in organisational changes and to pass on their experience through mentoring. To enable more autonomy, influence, and freedom within work at individual level, which was requested in all countries, a bottom-up management approach is once more suggested. According to Widell Blomé, Borell, Nilsson, and Håkansson [44], organisational preconditions and attitudes need to be considered when planning age management interventions. These kinds of organisational conditions may promote individual job crafting; thus optimizing work to the employees’ own abilities and energy [20,27]. Eaves, et al. [48] found that construction workers actively changed their work conditions to reduce challenging demands (e.g., safety risks), to make work easier, and to reduce musculoskeletal symptoms and increase overall health. This can be compared to Gaudart [49] who found that older assembly line workers developed strategies that enabled them to work at a steadier pace than their younger colleagues (e.g., by minimizing movements back and forth to collect gears) in order to protect them from physical strain. These examples of job crafting [28,50] can be compared to the respondents strategies found in this study, which included minor changes in the performance of tasks and to bring suggestions of improvements to the weekly quality meetings to reduce physical work load. Another job crafting activity found was to add meaning to one’s job assignments. To reframe what to think of one’s job in this way is one kind of cognitive crafting argued to be of particular value to older workers [51]. The perceived opportunities to job craft, however, differed among the respondents. Some said they had no opportunity to make any changes at all, whereas others were of a different opinion with a more positive mind set for making individual changes in work, in tasks, relations, and perception of work.

The survey did not include questions to cover the chrono-level. However, there are time-related aspects to consider when planning age management interventions in a global company. Firstly, the dimensions of ageing in the swAge-model interplay with the aspects found to be important to the blue-collar workers in this study. For example, the statutory retirement age differ between the three countries. This aspect is connected to chronological ageing and must be considered in a global company’s age management. Secondly, it may not be possible for companies themselves to affect trends and political decisions, but it might be important to adapt to, or consider them in the companies’ age management. One example is the statutory retirement age, currently rising in many European countries [52], as well as the fact that even though we live longer we do so with more chronic diseases, and not necessarily with a higher, or even maintained, work ability compared to previous generations. This is found especially among low-educated people [53]. Thirdly, the changing demographics with more older non-working people to provide for is perhaps one of the most obvious aspect to consider in age management strategies; ways to develop sustainable work settings that enable people to work longer with maintained health are needed. It is also important to consider the time factor when planning and implementing age management strategies within an organisation. For example, if a company chooses to educate their managers in age management, it will take time to educate all managers, as well as to implement the new knowledge in the company. There might also be a time difference in how fast the strategies can be adopted between the countries. In this study, the company already had a strategy for part time and early retirement through the local pension system in Sweden, which may offer better conditions for implementing certain age management strategies compared to the other countries.

To adapt the work to the individual workers, managerial decisions such as to enable part-time retirement, to customize work tasks, and to increase work autonomy can be effective ways to best make use of the competence of older workers [32,44].

### Method Discussion and Limitations

It must be considered that the respondents may have interpreted the content of the questions differently, for example, if they considered themselves to be ‘pushed’ to retirement by some aspects in a negative sense or if they were positively ‘pulled’ towards their own decisions about working or retiring. There may also have been differences in how the concept ‘prolonged work life’ was interpreted; it did not necessarily mean to prolong work life beyond statutory retirement age, but to work as long as possible.

There may be differences in the degree of respondents’ honesty and willingness to give information, depending on if the interviewer was someone from the company or an external person. In Sweden and the Netherlands there were also more than one interviewer, which may have had an effect on the data gathered, e.g., in terms of how comments were phrased. However, all the data was handled in the same manner when reported back to the first author of the article, and a close contact within the project teams during the entire study hopefully limited the effect on the results.

If the respondent had stated that the aspect was important, but not commented in what direction, the interview answers were used to understand in what way the aspect was considered as important. To be able to use both survey data and interview data is considered as a strength in the study design, although it is important to keep in mind that cultural differences may influence how appropriate the interview questions captured the essential opinions of the respondents. French blue-collar workers were not included in the pilot study, which can be seen as a weakness of the study design. If French respondents had been included in the pilot study, the survey and interview questions may have been more appropriate also to the French context and no aspects may have been removed from the French version of the survey.

The respondents were selected to provide us with the most genuine information; they were those who had retired most recently and those having the shortest amount of time left to retirement within the different production plants. In the planned study design, we hoped to include respondents that had left the company, or had been redeployed, due to sickness or physical issues. Those were however hard to track, and no respondent from that category was found. This may indicate ‘the healthy worker effect’; that the interviewees represented a healthier sample than in the society on average [54]. We also had a small number of women among the respondents (*n* = 6). The low number of female respondents limits us in making any assumptions about gender differences. Even though the responses from the women in our study did not indicate any notable trends from the male respondents, there might be other aspects related to female blue-collar workers’ situation than those appearing in this study.

The results of contributing aspects in this study are connected to the organisational context, and other known and unknown aspects in the area may not be covered. For example, an important health aspect in the physical work environment is vibrations. In our study, a rather low number of the respondents (8%) considered the aspect crucial to the decision about retirement, probably because most of the respondents did not consider themselves as exposed to vibrations. When asked about other possible aspects than the ones covered in the interview, comments were that there were none, and that work was not the most important factor to the decision. Instead health, family, and leisure time were said to be more important. Some said it was simply time to stop after a long work life.

## 5. Conclusions and Practical Implications

The results of this study contribute with a system perspective on age management, revealing aspects that a global company can affect, as well as some aspects on macro-, and chrono-levels that needs to be taken into account, when planning age management strategies. Both causes of early retirement and enablers of a prolonged work life were found on all levels; including individual-, organisational-, and societal levels.

The practical implications of this paper can contribute to the transition from work to retirement, from both employers’ and employees’ perspectives. For the employer this paper contributes with knowledge on what enables blue-collar workers to maintain in work, and how to facilitate this. From the employee perspective, the results can be used for raising their understanding and awareness of what factors might influence their willingness to prolong work life. As discussed above, this study found several factors influencing the retirement decisions among blue-collar workers, which cannot be solved separately. We also found differences among the respondents in the three different countries, but perhaps more importantly, there were differences between respondents within the same country and national settings. For example, in Sweden, some, but not all, respondents were able to retire early because of the local pension system. In all countries, there were also those who said they would like to retire with their health intact, but were not financially able to stop working. The results, above all, indicate that the different system levels interact and a system perspective is of great value when planning age management interventions in global organisations. Manufacturing companies that aim to enable blue-collar workers to work longer, and stay healthy whilst doing so, can thus benefit from using a system perspective, for example, the already established swAge-model [24], when planning and conducting age management interventions. Because of the individual differences among older workers, the possibility to craft their jobs, in a more autonomous setting, may be of particular importance. Further research, on how to conduct successful job crafting interventions for older blue-collar workers from a system perspective, is therefore suggested.

## Figures and Tables

**Table 1:** Pension systems and local agreements in the different countries.

	Sweden	The Netherlands	France
Statutory retirement age	61–67 In 2017, state pension was available from age 61, and guarantee pension was available from age 65. Age for state pension will rise from 62 in 2020, to 63 in 2023 and 64 in 2026.	65 in 2017, 66 in 2018 and 67 in 2021. From 2022, the state pension will be calculated according to year of birth and life expectancy.	62 Full state pension available from 62 years of age or more, if one has worked for 167 quarters for people born between 1958 to 1960; 168 quarters for people born between 1961 to 1963 and so on.
Effective (average) retirement age (men/women)	65/64 [39]	63/62 [39]	59/60 [39]
Local agreements at the company	Blue-collar workers aged 62 who has been employed for at least 30 years, or aged 63 and employed for at least 25 years, can retire with full occupational pension (compared to age 65). When turning 60, employees are entitled to part-time retirement.	When approaching retirement age, workers can use saved holidays to take one or more days off each week. When retiring before the calculated pension age, the occupational pension will pay out less accordingly.	Normal working time is 35 h per week. Blue-collar workers at the company work 37 h per week. Extra hours can currently be used as reduction of working time to start retirement earlier.

**Table 2:** Sample characteristics.

		SE	NL	FR	Total
Respondents	Men	24	36	34	94
	Women	5	1	0	6
	Total	29	37	34	100
Age	Range	59–69	57–67	56–62	56–69
	Average	63 (62.9)	65 (64.62)	58 (58.29)	62 (61.87)
	Std	2.41	2.06	1.96	3.46
Years of employment at Scania	Range	5–44	4–51	4–25	4–51
	Average	30 (29.6)	37 (36.92)	20 (20.47)	29 (29.2)
	Std	12.4	9.46	6.57	11.78
Work/retirement	Full-time working	20 (89%)	29 (78.4%)	30 (88.2%)	79 (79%)
	Part-time working	1 (3.4%)	2 (5.4%)	4 (11.8%)	7 (7%)
	Full-time retirement	8 (27.6%)	6 (16.2%)	0	14 (14%)

(SE = Sweden. NL = the Netherlands. FR = France).

**Table 3:** Factors and examples of items included in the survey.

Factors	Examples of Items (“Which of the Following Aspects are Crucial When You Consider Retirement?”)	References
Physical work environment	Heavy workload; Repetitive tasks; Disturbing noise	[1,4,5,6,7,8,9,10]
Psychosocial work environment	Demand and control of the work; Experienced work-related stress (e.g., due to lack of time or insufficient resources); Cooperation and possibility to get help when needed	[1,4,5,6,7,9,10,17]
Work organisation	Working hours and pauses; Setup of working shift and job rotation	[1,6,8,9,11,19,32]
Job task characteristics	Interesting job assignments; Having meaningful work tasks	[1,6,8,14,17,19]
Personal effort	To be content with your own effort; Commitment and attachment to the job and the company	[1,6,8,9,14,17,19]
Economic situation	Need to keep working to provide for your basic needs; How you consider national and local pension systems (e.g., generous or tough)	[1,9,14,16,17]
Health	Self-experienced physical and mental health; How you think your health will change during the following years	[1,8,9,12,13,14,17]
Attitude towards an older work force	How you experience the attitude towards an older workforce at your job; -among your family and friends	[1]
Personal factors	Having a spouse who is already retired; Wanting to spend more time with family and friends, or on leisure activities	[1,6,8,9,14,18]

**Table 4:** Most important findings and main differences between the countries.

System Level	Most Important Findings	Main Differences between the Countries
Macro	On the macro-level national pension systems were considered tough.	This was mainly found in Sweden and the Netherlands. In Sweden the local pension system still enabled workers to retire earlier, whereas workers in both France and the Netherlands said they needed to keep working to provide for their basic needs.
Meso	Setup of working shifts, and work distribution were the most prominent aspects on the meso-level.	Shift work, especially over night shifts, were considered tough by respondents in Sweden and the Netherlands. French respondents were more positive to shift work in general. Swedish respondents were more negatively affected by the work distribution than respondents in the other countries, where comments were that work enabled variation and recovery during the day.
Micro	The most positive aspects to prolong work life on the micro-level were social support from colleagues and superiors, and the atmosphere at work. Most negative aspects were physical work load, repetitive work tasks, and work related stress.	French respondents said the support from closest superior was lacking, and that the positive atmosphere at work had decreased. Thus, both aspects had a negative impact on their retirement decision, in contrast to the respondents in Sweden and the Netherlands.
Individual	Self-experienced physical and mental health, expected future health, and to spend more time with family and friends, were the most important aspects to retirement in all countries. The economic situation was the most prominent factor to prolong work life.	The local pension system, as mentioned above, was the only thing that differed between the countries on this level, which enabled Swedish respondents to retire earlier.

**Table 5:** Result of the survey; percentages of respondents considering the aspects as either contributing to retirement or to a prolonged work life, or as having no effect on the decision. Percentages is presented within each country and in total.

	Sweden			The Netherlands			France			Total		
	No Effect	To Retirement	To A Prolonged Work Life	No Effect	To Retirement	To A Prolonged Work Life	No Effect	To Retirement	To A Prolonged Work Life	No Effect	To Retirement	To A Prolonged Work Life
**Physical Work Environment**												
ASPECT												
Physical workload	58.6	34.5	6.9	56.8	35.1	8.1	79.4	20.6	0	65	30	5
Ergonomic tools and adequate safety equipment	86.2	10.3	3.4	83.8	0	16.2	79.4	2.9	17.6	83	4	13
Repetitive work	75.9	20.7	3.4	83.8	10.8	5.4	70.6	29.4	0	77	20	3
Working position/posture	82.8	17.2	0	100	0	0	79.4	20.6	0	88	12	0
Variation and recovery	72.4	20.7	6.9	48.6	18.9	32.4	73.5	8.8	17.6	64	16	20
Lightning	96.6	3.4	0	97.3	2.7	0	91.2	8.8	0	95	5	0
Disturbing noise	86.2	13.8	0	100	0	0	88.2	11.8	0	92	8	0
Vibrations	89.7	10.3	0	97.3	2.7	0	88.2	11.8	0	92	8	0
Temperature	93.1	6.9	0	97.3	2.7	0	94.1	5.9	0	95	5	0
High risk of work-related injuries	89.7	10.3	0	100	0	0	79.4	20.6	0	90	10	0
Exposed to chemicals	93.1	6.9	0	100	0	0	94.1	5.9	0	96	4	0
**Psychosocial work environment**												
ASPECT												
Demands and control in work	89.7	10.3	0	51.4	27	21.6	67.6	26.5	5.9	68	22	10
Work-related stress	51.7	48.3	0	81.1	18.9	0	61.8	38.2	0	66	34	0
Clear organisational goals	96.6	3.4	0	97.3	2.7	0	88.2	8.8	2.9	94	5	1
Social support from colleagues	89.7	3.4	6.9	40.5	2.7	56.8	67.6	17.6	14.7	64	8	28
Support from closest superior	79.3	6.9	13.8	43.2	10.8	45.9	64.7	26.5	8.8	61	15	24
The atmosphere and comfort at work	72.4	6.9	20.7	29.7	5.4	64.9	47.1	38.2	14.7	48	17	35
Cooperation and possibility of getting help when needed	82.8	3.4	13.8	64.9	2.7	32.4	70.6	26.5	2.9	72	11	17
Relationship with co-workers and superior(s)	75.9	10.3	13.8	51.4	2.7	45.9	70.6	23.5	5.9	65	12	23
**Work organisation**												
ASPECT												
Work distribution during the day	89.7	10.3	0	48.6	13.5	37.8	67.6	11.8	20.6	67	12	21
Working hours and pauses	82.8	10.3	6.9	81.1	8.1	10.8	70.6	14.7	14.7	78	11	11
Setup of working shifts	69	20.7	10.3	37.8	48.6	13.5	79.4	11.8	8.8	61	28	11
Setup of job rotation	82.8	13.8	3.4	86.5	2.7	10.8	67.6	8.8	23.5	79	8	13
Work station in order	96.6	3.4	0	97.3	2.7	0	76.5	17.6	5.9	90	8	2
Clear job description	93.1	3.4	3.4	97.3	2.7	0	79.4	5.9	14.7	90	4	6
Knowing who to ask if needed	100	0	0	97.3	2.7	0	Question was not used in France			98.5 †	1.5 †	0 †
Correct and functioning information flow	89.7	10.3	0	97.3	2.7	0	88.2	5.9	5.9	92	6	2
Management approach	82.8	13.8	3.4	67.6	18.9	13.5	67.6	26.5	5.9	72	20	8
Experienced development opportunities	86.2	6.9	6.9	97.3	2.7	0	88.2	8.8	2.9	91	6	3
**Job task characteristics**												
ASPECT												
Interesting and meaningful job assignments	65.5	3.4	31	86.5	2.7	10.8	52.9	23.5	23.5	69	10	21
Influence and responsibility for others as well as for the work procedures	72.4	3.4	24.1	89.2	2.7	8.1	Question was not used in France			81.8 †	3 †	15.2 †
Suitable competence to conduct job assignments	86.2	0	13.8	94.6	2.7	2.7	67.6	11.8	20.6	83	5	12
**Personal effort**												
ASPECT												
One’s professional identity	75.9	6.9	17.2	89.2	2.7	8.1	73.5	5.9	20.6	80	5	15
To be content with one’s own effort	62.1	3.4	34.5	91.9	2.7	5.4	52.9	14.7	32.4	70	7	23
Appreciation from others on work conducted	65.5	6.9	27.6	83.8	0	16.2	73.5	8.8	17.6	75	5	20
Meaningful job assignments and mission	65.5	3.4	31	81.1	2.7	16.2	85.3	2.9	11.8	78	3	19
Being able to use one’s competence, education, and experience in a relevant way	72.4	6.9	20.7	91.9	2.7	5.4	73.5	14.7	11.8	80	8	12
Commitment and attachment to the job and the company	72.4	3.4	24.1	89.2	2.7	8.1	67.6	11.8	20.6	77	6	17
**Economic situation**												
ASPECT												
Need to keep working to provide for one’s basic needs	89.7	3.4	6.9	29.7	2.7	67.6	50	2.9	47.1	54	3	43
Choose to work some extra years to raise one’s future pension	75.9	0	24.1	94.6	0	5.4	67.6	2.9	29.4	80	1	19
Pension is not enough to provide the life-style one is used to	82.8	6.9	10.3	78.4	2.7	18.9	82.4	2.9	14.7	81	4	15
Providing for others in household	93.1	0	6.9	89.2	2.7	8.1	91.2	2.9	5.9	91	2	7
Pension enough to provide without income	58.6	41.4	0	91.9	8.1	0	85.3	0	14.7	80	15	5
Having another income	100	0	0	97.3	2.7	0	100	0	0	99	1	0
National and local pension systems considered tough or generous	72.4	6.9	20.7	78.4	8.1	13.5	97.1	0	2.9	83	5	12
**Health**												
ASPECT												
Self-experienced physical and mental health	37.9	41.4	20.7	45.9	40.5	13.5	41.2	52.9	5.9	42	45	13
Feeling good or feeling bad in general	62.1	27.6	10.3	75.7	16.2	8.1	Question was not used in France			69.7 †	21.2 †	9.1 †
Sense of coherence	82.8	17.2	0	100	0	0	Question was not used in France			92.4 †	7.6 †	0
In control of matters that affect one’s life	82.8	17.2	0	100	0	0	67.6	26.5	5.9	84	14	2
Confronted with life crisis	86.2	13.8	0	83.8	10.8	5.4	79.4	17.6	2.9	83	14	3
Expected future health	48.3	44.8	6.9	43.2	56.8	0	85.3	14.7	0	59	39	2
**Attitude towards an older work force**												
ASPECT												
Attitude at one’s work place	79.3	6.9	13.8	91.9	5.4	2.7	91.2	8.8	0	88	7	5
Attitude among family and friends	93.1	0	6.9	100	0	0	97.1	0	2.9	97	0	3
Attitude in the society	86.2	6.9	6.9	97.3	2.7	0	97.1	2.9	0	94	4	2
Attitude in the culture	100	0	0	97.3	2.7	0	Question was not used in France			98.5 †	1.5 †	0
Affected by stereotyping	96.6	3.4	0	100	0	0	97.1	2.9	0	98	2	0
Experience of ageism	93.1	6.9	0	97.3	2.7	0	Question was not used in France			95.5 †	4.5 †	0 †
**Personal factors**												
ASPECT												
Partner’s retirement	82.8	3.4	13.8	86.5	13.5	0	85.3	11.8	2.9	85	10	5
Social life connected to work	100	0	0	100	0	0	100	0	0	100	0	0
More time for family, friends, and leisure activities	41.4	58.6	0	59.5	40.5	0	73.5	26.5	0	59	41	0
More time for a spare-time job or volunteer work	79.3	17.2	3.4	100	0	0	79.4	20.6	0	87	12	1

† French respondents excluded from the total.
